# Landmark-guided versus modified ultrasound-assisted Paramedian techniques in combined spinal-epidural anesthesia for elderly patients with hip fractures: a randomized controlled trial

**DOI:** 10.1186/s12871-020-01172-x

**Published:** 2020-09-28

**Authors:** Bo Qu, Luying Chen, Yuling Zhang, Mengting Jiang, Caineng Wu, Wuhua Ma, Yuhui Li

**Affiliations:** 1 Guangzhou University of Chinese Medicine, Guangzhou, 510405 Guangdong China; 2 Department of Anesthesiology, The First Affiliated Hospital of Guangzhou University of Chinese Medicine, Guangzhou, 510405 Guangdong China

**Keywords:** Combined spinal-epidural anesthesia, Ultrasonography, Aged, Hip fractures

## Abstract

**Background:**

Combined spinal-epidural (CSE) anesthesia is considerably challenging for elderly patients with hip fractures due to spine degeneration and limitations in positioning. This study aimed to investigate the ability of a modified preprocedural ultrasound-guided technique to improve the success rate and efficacy of CSE anesthesia for elderly patients with hip fractures.

**Methods:**

This prospective, single-blinded, parallel-group randomized controlled trial included 80 patients (aged ≥65 years) who were scheduled for elective hip fracture surgery with CSE anesthesia. Patients were randomly allocated into landmark group (*n* = 40) or the ultrasound group (*n* = 40). The primary outcome was first-pass success rate. Secondary outcomes included first-attempt success rate; number of needle insertion attempts; number of needle passes; locating, puncture, and total time; level of block; procedural adverse reactions and postoperative complications; and patient satisfaction score. Patients were blinded to group allocation.

**Results:**

Eighty patients completed the study and were included in the final analysis. The first-pass success rates for the landmark and ultrasound groups were 20 and 70%, respectively (*P* < 0.001). The first-attempt success rates in the landmark and ultrasound groups were 42.5 and 85%, respectively (*P* < 0.001). The median number of attempts was lower in ultrasound-assisted group (1 [1]) than landmark-guided group (2 [1, 2]), *P* < 0.001). The median number of needle passes was lower in ultrasound group (1 [1, 2]) than in landmark-guided group (3 [2, 4], *P* < 0.001). The locating time (*P* < 0.001) and total time (*P* = 0.001) were longer in the ultrasound group, while puncture time was shorter (*P* = 0.003). No significant difference was found regarding the incidence of adverse reactions and complications. More patients in the ultrasound group had a high satisfaction score of 4–5 (*P* = 0.007). Interestingly, subgroup analysis demonstrated benefits for ultrasound in patients with scoliosis.

**Conclusions:**

Modified ultrasound-assisted CSE anesthesia increases first-pass and first-attempt success rates, and reduces needle insertion attempts, passes, and puncture time for elderly patients with hip fracture, especially those with scoliosis. This technique improves patient satisfaction and warrants consideration for application in clinical practice.

**Trial registration:**

Chinese Clinical Trial Register (identifier, ChiCTR1900020819; date of registration, January 20, 2019).

## Background

Hip fracture is the second leading cause of hospitalization in the elderly population, its incidence is increasing with age [1–3]. Compared with general anesthesia, patients who receive combined spinal-epidural (CSE) anesthesia for hip surgery have a lower 30-day mortality [4, 5] and shorter hospital stays [1, 4, 6]. Traditional CSE anesthesia relied on the palpation of surface landmarks to identify the intervertebral levels; however, the possible occurrence of spine degeneration, supraspinous and interspinous ligament calcification, narrowing of intervertebral space, lumbar scoliosis, and deformities may make the identification of the intervertebral space unreliable and cause difficulties in needle insertion [7–10] In addition, the limitation in body positioning in patients with hip fracture may limit the opening of intervertebral space, and make the puncture challenging in traditional landmark-guided technique [11, 12].

The ultrasound-assisted CSE anesthesia technique provides improved precision and efficacy, overcoming the technical difficulties of performing neuraxial blocks [13–17] for obese [18, 19], obstetric [20–23], and aged patients [12, 24, 25], as well as patients with difficult-to-detect and abnormal anatomical surface landmarks [9, 26]. However, few studies have focused on ultrasound-assisted CSE anesthesia in elderly patients who have difficulty achieving optimal body positioning. The paramedian technique is the preferred choice of CSE anesthesia for the elderly. However, its success requires proper cephalad [27] and medial needle angulation [28]. Previous studies have determined the optimal needle insertion point and depth via ultrasonography; however, the ideal needle angulation has not been investigated to date [24, 29]. Furthermore, while the ultrasound-assisted central neuraxial block has been conventionally applied in spinal anesthesia with either a midline [22] or paramedian approach [24, 29, 30], and in CSE anesthesia with a midline approach [23], few studies have investigated the use of a paramedian approach in CSE anesthesia.

The current study aimed to investigate whether the ultrasound-assisted paramedian CSE anesthesia technique, modified with suggested needle insertion angulations and a more caudad needle insertion point, can contribute to an improved first pass success rate than conventional landmark-guided paramedian technique in elderly patients with hip fractures.

## Methods

### Study design and participants

**Fig. 1:**
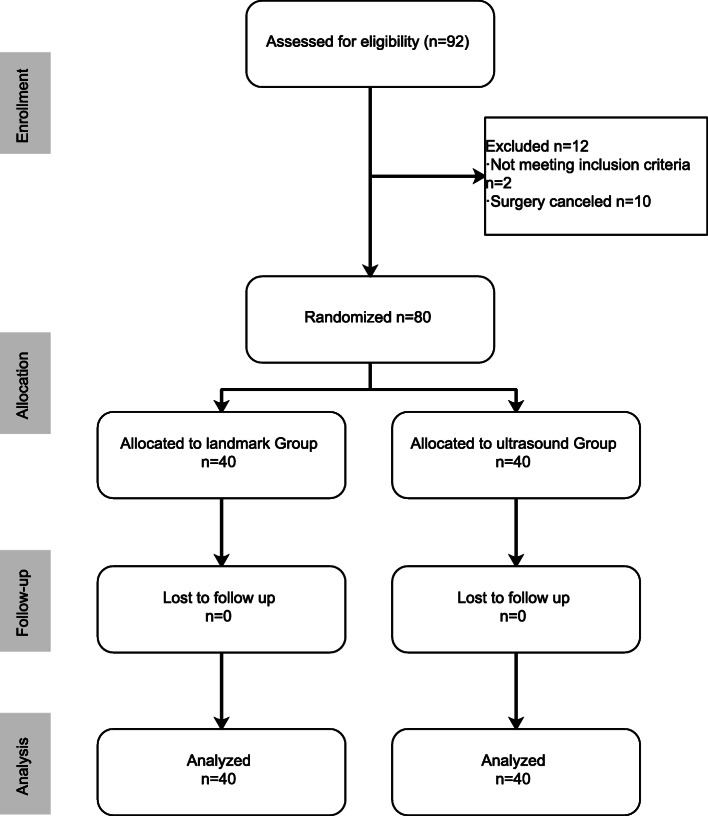
Consolidated Standards of Reporting Trials diagram showing the progress of patients through the study

This prospective, randomized controlled trial was approved by the hospital’s Institutional Review Board (Ethics Committee of The First Affiliated Hospital of Guangzhou University of Chinese Medicine; Y [2019]042; February 11, 2019) and written informed consent was obtained from all patients participating in the trial. This study was registered prior to patient enrolment at the Chinese Clinical Trial Register (identifier, ChiCTR1900020819; principal investigator, Y.L.; date of registration, January 20, 2019). The trial was performed from February 2019 to September 2019 in The First Affiliated Hospital of Guangzhou University of Chinese Medicine, Guangzhou, China, and adhered to the applicable Consolidated Standards of Reporting Trials guidelines (Fig. 1).

A total of 80 patients were recruited. The inclusion criteria comprised (1) patients who were scheduled to receive CSE anesthesia for elective hip fracture surgery; (2) age ≥ 65 years; (3) body mass index (BMI) ≤ 30 kg/m^2^; and (4) an American Society of Anesthesiologists (ASA) classification of I to III. Exclusion criteria are as follows (1) severe cardiopulmonary diseases; (2) a contraindication to CSE anesthesia (e.g., coagulopathy, hypovolemia, raised intracranial pressure, infection in puncture area, allergy to local anesthetics, or lack of cooperativity); and (3) a history of lumbar surgery.

### Randomization

The patients were randomized (using a computer-generated randomized number table) to receive CSE anesthesia using either a landmark-guided technique (*n* = 40) or an ultrasound-assisted technique (*n* = 40). The allocation of patients was determined by sequentially numbered, sealed envelopes after the patients were moved into the operating room. During the procedure, patients were blinded to group allocation.

### Procedures

Three anesthesiologists conducted the trial, and each had previously performed more than 40 ultrasound-assisted neuraxial blocks and ample experiences (> 15 years in average) in conducting CSE anesthesia. In the landmark group, ultrasound and CSE anesthesia were performed by distinct operators, while the whole procedure in the ultrasound group was performed by the same operator.

After the patients were moved to the operating room, routine monitoring (non-invasive blood pressure, 3-lead electrocardiogram, oximetry) and face mask oxygen at a flow rate of 1–2 L/min were applied, and peripheral intravenous access was established. During the whole procedure, no sedative was administered. An ultrasound-guided fascia iliaca compartment block was performed with 20 mL of 0.375% ropivacaine to reduce pain for all patients [31, 32]. After 15 min, the patient was assisted in assuming a lateral decubitus position with the fracture side up. In both groups, the anesthesiologists palpated the surface landmark and graded the ease of palpation using a 3-point scale (easy, moderate, and difficult) as described in a previous study [25].

For the landmark group, the procedure was done following these three steps:

- Identification of the needle insertion point. The needle insertion point was marked on the skin by traditional palpation. The first anesthetist subsequently left the operating room.
- Ultrasound scan. A portable ultrasound machine (Konica Minolta, SONIMAGE HS1, Japan) with a low frequency (2–5 MHz) curved array probe with a depth of 8 cm was used. Due to safety concerns, a second anesthesiologist conducted an ultrasound to check if the skin mark was above the L1-L2 interlaminar space; if so, the anesthetist was required to perform CSE anesthesia at a lower interlaminar space [23]. Ultrasound images were saved.
- Administration of CSE anesthesia. CSE anesthesia was performed by the first anesthesiologist, using the paramedian approach.

**Fig. 2:**
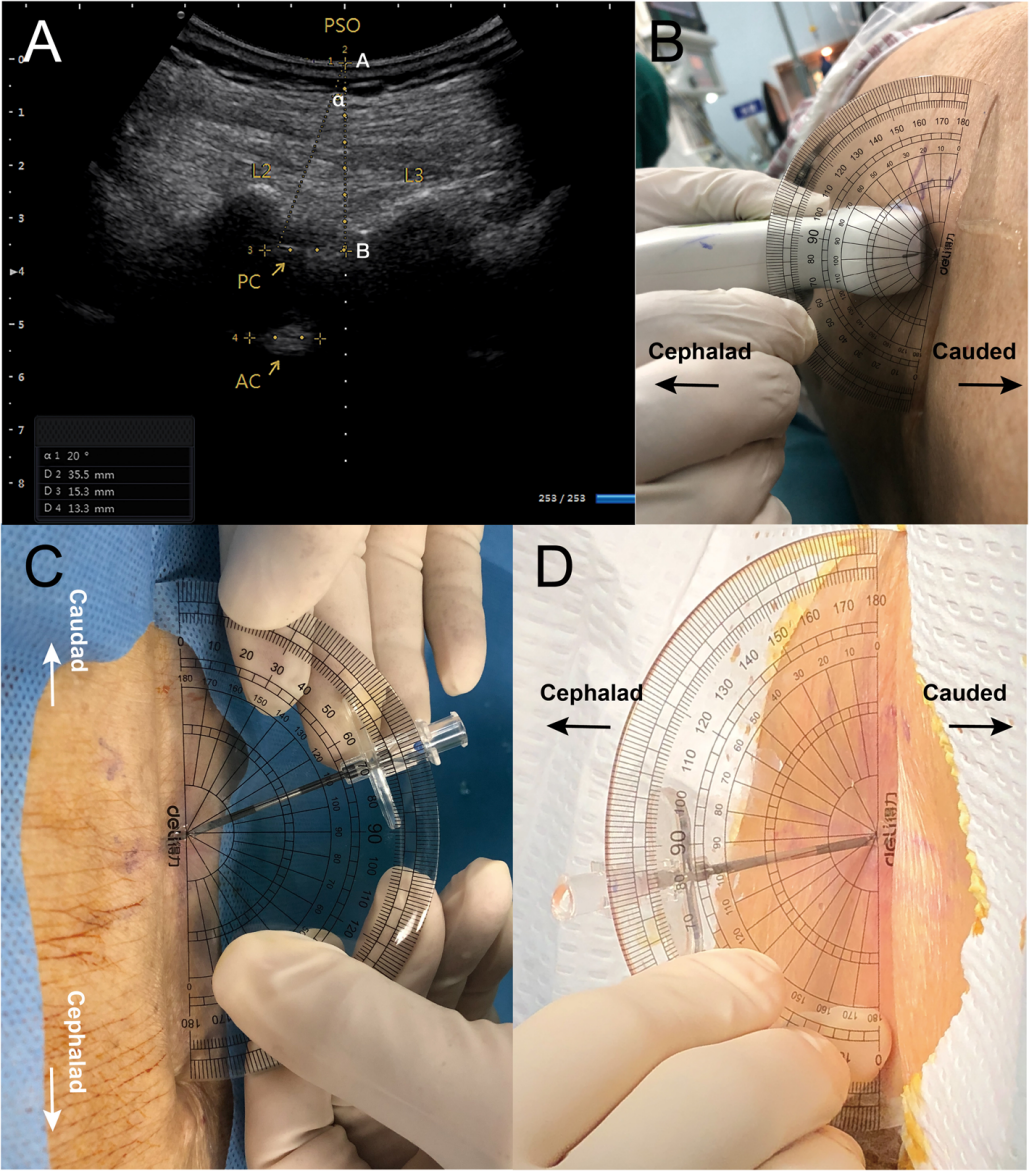
Measurement and application of the needle angle. **a** An ultrasound image from the paramedian sagittal oblique (PSO) view, which shows the L2-L3 interlaminar space, posterior complex (PC), and anterior complex (AC). ∠α is the measured maximum cephalad angle of the needle. A to B is the distance from the skin to the posterior complex. **b** Measuring the medial angle with a 180° protractor during the paramedian approach, which is the tilting angle of the probe to the median plane. **c** An aseptic 180° protractor was used to assist the cephalad needle insertion angle. **d** After a successful puncture, the actual medial angle was measured with an aseptic 180° protractor

For the ultrasound group, the entire procedure was carried out using the following six steps:

- Marking of the midline. The probe was placed at the transverse midline (TM) plane for the evaluation of spine anatomy. The probe was tilted to obtain optimal ultrasound images. Midpoints of the long edge of the ultrasound probe were marked as the midline of the spine.
- Identification of the interlaminar space. The probe was placed at the parasagittal oblique (PSO) plane, 1–2 cm to the midline. The scan was performed upwards from the sacrum; the L5-S1 to L2-L3 interlaminar spaces were identified successively by the “counting-up” approach. The primary and secondary choice of interlaminar space for puncture were determined by the ultrasound image quality and the length of the anterior/posterior complex.
- Identification of the needle insertion point. The probe was adjusted to achieve the best ultrasound image at the determined interlaminar space. Then, the upper edge of the inferior laminar was placed at the center of the ultrasound screen. Skin marks were made at the midpoints of the long and short borders of the probe. The intersection of two connecting lines indicated the needle insertion point.
- Measurement of the suggested insertion angles. The built-in tool in the ultrasound unit was used to measure the maximum cephalad angle (∠α in Fig. 2a) between (1) the connecting line from the insertion point to the far end of the posterior complex and (2) the midline of the ultrasound screen; 1/2 ∠α was the suggested cephalad angle. The probe’s tilt to the median plane indicated the medial angle (∠β), and was measured using a 180° protractor (Deli, Shanghai, China) (Fig. 2b).
- Measurement of the needle insertion depth. The distance from the insertion point to the posterior complex, which was the presumed minimum insertion depth, was measured using the ultrasound clipper tool (Fig. 2a).
- Administration of CSE anesthesia. CSE anesthesia was conducted using the paramedian technique according to the marked insertion point, suggested insertion angles, and presumed depth. After the needle reached the subcutaneous tissue and became stable, a low temperature plasma sterilized protractor (Deli, Shanghai, China) was used to correct the needle insertion angle (Fig. 2c). When the puncture was successful, the actual needle insertion angles (cephalad and medial) were measured (Fig. 2d).

In both groups, an aseptic technique was strictly applied throughout the entire process. CSE anesthesia was performed using a needle-through-needle approach, with a 25/16-gauge CSE kit (Kindao Interventional Medical Co., Ltd., Guangzhou, China). When the backflow of clear cerebrospinal fluid was observed, 0.5% ropivacaine (9.75–12.75 mg) was injected. Then, a 20-gauge multi-orifice epidural catheter (Kindao Interventional Medical Co., Ltd., Guangzhou, China) was inserted through the Touhy needle, up to 5 cm into the epidural space. If three attempts failed, the secondary interlaminar space was used. If attempts at two different interlaminar spaces failed, an alternative technique was allowed (palpation, ultrasound guidance, midline approach, another anesthetist). In the event that the alternative technique failed, general anesthesia was induced.

The patient satisfaction score was rated using a 5-point scale (from 1: completely dissatisfied to 5: completely satisfied) after anesthesia [23]. The block level was tested by loss of cold sensation, 15 min after anesthesia. The quality of the ultrasound image was assessed as good (the posterior complex and anterior complex were both visible), moderate (either the posterior complex or anterior complex was visible), or poor (neither the posterior nor anterior complex was visible) [24, 29, 33]. The discrepancy (Δ) between the suggested and actual angle was classified as accurate (0° ≤ Δ ≤ 5°), acceptable (5° < Δ ≤ 10°), or inaccurate (Δ > 10°). During the entire procedure, data were recorded by a research assistant; for all measurements, the mean of three readings was calculated. A postoperative follow-up was conducted within 48 h after the surgery.

### Study outcomes

The primary outcome in this study was the first-pass success rate of CSE anesthesia. A first-pass success was defined as the needle reaching the subarachnoid space within a single insertion attempt, without redirection.

Secondary outcomes were as follows:

- First-attempt success rate: defined as the needle reaching the subarachnoid space within a single insertion attempt and allowing redirection.
- Number of needle insertion attempts: each skin puncture was considered as a separate attempt.
- Number of needle passes: total number of insertion attempts and needle redirections.
- Locating time: the time from when the operator touched the patient’s skin to the marking of the insertion point on the skin (landmark group), and the time from when the probe was placed on the skin to the marking of the insertion point (ultrasound group).
- Puncture time: interval between the contact of the skin with the Touhy needle, and the observation of cerebrospinal fluid from the spinal needle.
- Total time: the sum of the locating time and puncture time.
- Level of block: measured by testing the loss of cold sensation.
- Procedural adverse reactions: radicular pain, bloody tap, unintentional dural puncture.
- Postoperative complications: including paresthesia, backache, and post-dural puncture headache.
- Patient satisfaction score: 1 (completely dissatisfied), 2 (dissatisfied), 3 (moderate), 4 (satisfied), 5 (completely satisfied). It was defined as the overall comfort level the patients experienced during the procedure, which includes (i) the back pain the patients felt, (ii) radicular pain the patient felt (iii) the discomfort due to the repositioning after a failed needle insertion and (iv) the overall anxiety or fear the patients felt. One negative response to these situations will deduct the satisfaction score by one point.

### Statistical analysis

The sample size was calculated using PASS software Version 15.0 (NCSS, Kaysville, USA). Based on our pilot study, the first-pass success rates in patients using the conventional palpation and ultrasound-assisted technique were 22 and 59%, respectively. With an α error of 5% and a *β* error of 10% (90% power), a sample size of 35 patients per group was required. We increased the target sample size to 40 patients per group to allow for dropouts.

Data were analyzed using SPSS 25.0 (IBM Corporation, NY, USA). Continuous data were tested for normality using the Kolmogorov-Smirnov test. Normally distributed data (mean ± standard deviation [SD]) were compared using the Student’s t-test. Non-normally distributed data (median [interquartile range]) were compared using the Mann-Whitney U test. Categorical variables were presented as n (%) and were compared using the χ2 test or Fisher’s exact test. The primary outcome (first-pass success rate) was compared using the χ2 test. Spearman’s rank correlation was used to determine the relationship between the presumed minimum needle insertion depth and actual insertion depth. For the differences in success rates for a selected number of passes and attempts between two groups, 95% confidence intervals (CI) were calculated. A two-tailed *P* < 0.05 was considered statistically significant.

Pre-specified sub-group analysis was conducted to investigate the effect of scoliosis to the first-pass success rate, number of needle passes and needle insertion attempts, locating time, puncture time, total time and patient satisfaction. Sub-group analysis was performed for all 12 patients with scoliosis, 6 patients in each group.

## Results

From February to September 2019, 92 elderly patients were recruited and assessed for eligibility. Eighty patients, aged 82.8 ± 6.8 years, were included for random allocation to the landmark (*n* = 40) or ultrasound (*n* = 40) group (Fig. 1). No data were missing, and no patients were lost to follow-up. The reasons for the 12 exclusions were that patients did not meet the inclusion criteria (*n* = 2), or surgery was canceled by the surgical department (*n* = 10).

There were no significant differences between the groups for baseline characteristics (Table 1). A significantly higher first-pass success rate (70% vs. 20%) and success rate within two passes (82.5% vs. 40%) were achieved in the ultrasound group vs. the landmark group (both *P* < 0.001; Table 2). The first attempt success rate in the ultrasound group was twice higher than that in the landmark group (85% vs. 42.5%, *P* < 0.001). However, no difference between the two groups was found for the success rate within two attempts (*P* = 0.264). A significantly lower median number of needle attempts and passes were achieved in the ultrasound group (both *P* < 0.001; Table 2).

**Table 1:** Patient characteristics

	Landmark-guided group *n* = 40	Ultrasound-assisted group *n* = 40	***P***-value
Age (y)	82.3 ± 7.1	83.3 ± 6.7	0.549
Height (cm)	156.7 ± 7.0	156.9 ± 7.2	0.925
Weight (kg)	50.6 ± 8.4	53.2 ± 10.1	0.203
BMI (kg/m^2^)	20.6 ± 3.0	21.6 ± 3.6	0.176
Sex (male/female)	7/33	10/30	0.412
ASA Classification			0.502
I	0 (0%)	0 (0%)	
II	22 (55%)	19 (47.5%)	
III	18 (45%)	21 (52.5%)	
Degree of back curvature			0.635^a^
Backward	4 (10%)	2 (5%)	
None	33 (82.5%)	36 (90%)	
Forward	3 (7.5%)	2 (5%)	
Scoliosis			1.000
Positive	6 (15%)	6 (15%)	
Negative	34 (85%)	34 (85%)	
Ease of landmark palpation			0.654^a^
Easy	34 (85%)	31 (77.5%)	
Moderate	5 (12.5%)	7 (17.5%)	
Difficult	1 (2.5%)	2 (5%)	

Data are presented as mean ± SD or n (%)

*Abbreviations: BMI* Body mass index, *SD* Standard deviation, *ASA* American Society of Anesthesiologists

^a^Fisher’s exact test

**Table 2:** Comparison of procedure related data

	Landmark- guided group *n* = 40	Ultrasound- assisted group *n* = 40	***P*** value	95% CI of differences (%)
First pass success, n (%)	8 (20)	28 (70)	< 0.001	(31.1 to 68.9)
Success within 2 passes, n (%)	16 (40)	33 (82.5)	< 0.001	(23.3 to 61.7)
First attempt success, n (%)	17 (42.5)	34 (85)	< 0.001	(23.6 to 61.4)
Success in 2 attempts, n (%)	34 (85)	38 (95)	0.264^a^	(−3 to 23)
Number of attempts	2 [1 to 2]	1 [1 to 1]	< 0.001	
Number of passes	3 [2 to 4]	1 [1 to 2]	< 0.001	
Locating time(s)	32.5 [21.3 to 40.8]	337.5 [300.0 to 403.8]	< 0.001	
Puncture time(s)	320.0 [223.3 to 583.0]	227.5 [170.0 to 340.0]	0.003	
Total time(s)	440.3 ± 240.1	608.2 ± 196.9	0.001	
Patients’ satisfaction;4–5	26 (65.0%)	36 (90.0%)	0.007	(7.5 to 42.5)

Data are presented as mean ± SD, median [interquartile range] or n (%)

*Abbreviations: SD* standard deviation, *CI* confidence interval

^a^Continuity correction

Compared with the landmark group, the locating time was much longer while the puncture time was shorter in the ultrasound group. A longer total time for CSE anesthesia was required in the ultrasound group. More patients rated their satisfaction of the CSE anesthesia as 4 or 5 in the ultrasound group (90% vs. 65%, *P* = 0.007, Table 2).

Discrepancies between suggested and actual angles are presented in Table 3. In terms of the cephalad angle, the actual cephalad angle exceeded the measured maximum angle in five cases. A total of 70% cases reached the “accurate” level. For the medial angle, 80% cases reached the “accurate” level.

**Table 3:** Angulation information obtained by ultrasonography

	Number of views
Comparison between actual and maximum cephalad angle	
actual angle ≤ maximum angle	35 (87.5%)
actual angle > maximum angle	5 (12.5%)
Cephalad angle discrepancy	
accurate 0° ≤ Δ^a^ ≤ 5° n (%)	28 (70%)
acceptable 5° < Δ ≤ 10° n (%)	7 (17.5%)
inaccurate Δ > 10° n (%)	5 (12.5%)
Medial angle discrepancy	
accurate 0° ≤ Δ ≤ 5° n (%)	32 (80%)
acceptable 5° < Δ ≤ 10° n (%)	6 (15%)
inaccurate Δ > 10° n (%)	2 (25%)

^a^Δ indicates the discrepancy between the suggested and actual angles

In all cases, the width of the posterior complex was 0.94 ± 0.22 cm, and that of the anterior complex was 1.24 ± 0.31 cm. The minimum needle insertion depth (measured through ultrasound imaging) had a certain correlation with the actual insertion depth (*r* = 0.514, *P* < 0.001).

A significant difference was found in the interspace level of the puncture between the two groups (*P* = 0.036; Table 4). The T8 or T10 dermatome level could be reached in all cases, and no significant difference was found between the two groups (*P* = 0.251; Table 4). In terms of procedural adverse reactions and postoperative complications, no significant differences were found between the groups. There were no occurrences of paresthesia, backache, or post-dural puncture headache. No patients were converted to general anesthesia in either group. In the landmark group, two patients were converted to alternative techniques, however, the difference was not significant between the two groups (*P* = 0.494, Table 5). In terms of the quality of the ultrasound images, more images of good quality were obtained in PSO views than in TM views (Additional Table 1).

**Table 4:** Interspinous level of successful puncture and block level

	Landmark- guided group *n* = 40	Ultrasound- assisted group *n* = 40	***P*** value
Interspace level of successful puncture n (%)			0.036
L2/L3	10 (25%)	19 (47.5%)	
L3/L4	30 (75%)	21 (52.5%)	
Peak dermatome level n (%)			0.251
T8	13 (32.5%)	18 (45%)	
T10	27 (67.5%)	22 (55%)	

Data are presented as n (%)

(Table 5) A subgroup analysis was conducted for 12 patients with scoliosis (Table 6). The first-pass success rate was 83.8% in the ultrasound group, and 0% in the landmark group (*P* = 0.015). Fewer attempts (*P* = 0.022) and needle passes (*P* = 0.016) were achieved in the ultrasound group. The locating time was longer in the ultrasound group (*P* = 0.004), while the puncture time was shorter (*P* = 0.043). The total time (*P* = 0.659) and patient satisfaction score (*P* = 0.061) were not significantly different between the two groups.

**Table 5:** Procedural adverse reactions and postoperative complications

	Landmark group (***n*** = 40)	Ultrasound group (***n*** = 40)	***P*** value^**a**^
Radicular pain	2 (5.0%)	2 (5.0%)	1
Bloody tap	3 (7.5%)	1 (2.5%)	0.615
Unintentional dural puncture	2 (5.0%)	1 (2.5%)	1
Backache	0 (0%)	0 (0%)	–
Post-dural puncture headache	0 (0%)	0 (0%)	–
Paresthesia	0 (0%)	0 (0%)	–
Alternative technique	2 (5.0%)	0 (0%)	0.494
Conversion to general anesthesia	0 (0%)	0 (0%)	–

Data are presented as n (%)

^a^Fisher’s exact test

**Table 6:** Subgroup analysis for patients with scoliosis

	Landmark- guided group *n* = 6	Ultrasound- assisted group *n* = 6	***P*** value	95% CI of differences (%)
First pass success, n (%)	0 (0.0%)	5 (83.3%)	0.015^a^	(71.7, 94.9)
Number of attempts	2 [1 to 5]	1 [1 to 1]	0.022	
Number of passes	3.5 [2.75 to 12.5]	1 [1 to 1.75]	0.016	
Locating time(s)	40.0 [33.75 to 45.75]	405.0 [256.75 to 466.25]	0.004	
Puncture Time(s)	535.33 ± 185.24	272.50 ± 206.80	0.043	
Total time(s)	576.33 ± 180.21	641.17 ± 298.78	0.659	
Satisfaction; 4–5	2 (33.3%)	6 (100.0%)	0.061^a^	(52.1, 81.3)

Data are presented as mean ± SD, median [interquartile range] or n (%)

*Abbreviations: SD* standard deviation, *CI* confidence interval

^a^Fisher’s exact test

## Discussion

The current study shows that in comparison with the landmark-guided technique, the ultrasound-assisted technique had a higher first-pass and first-attempt success rate, fewer needle passes and insertion attempts, and a shorter puncture time; this improved the efficacy of CSE anesthesia, as well as patient satisfaction.

CSE anesthesia was applied to reduce the dose of local anesthetic in spinal anesthesia; this lowered the risk of unstable hemodynamic conditions among the elderly patients, who had a high prevalence of underlying diseases. Epidural cathetering was applied to ensure an adequate block level during the surgery, and maintenance of postoperative analgesia [34]. Ultrasound scan was conducted in all patients for three reasons. Firstly, it is for safety concern, even the occurrence of L1-L2 is very unlikely, this procedure can ensure the needle insertion point is below L1-L2, which is endorsed by A. Chin et al. [23]. Secondly, the ultrasound scan can provide more data for evaluation of the ultrasound image quality. Lastly, since the patients were blinded to the procedure, ultrasound scan in both groups could reduce the procedural difference which may influence the patients’ satisfaction rating.

Compared with previous studies [24, 25], the subjects in the current study had a higher mean age and lower lumbar curvature ability; furthermore, patients with scoliosis were also included. Thus, puncture was relatively more difficult in the present study. Additionally, although total duration of the procedure was significantly longer in the ultrasound group, more time was used in ultrasound scan which will not discomfort the patients. In landmark-guided group, more time was used for puncture or repositioning, which may reduce the patients’ satisfaction. Overall, we believe at the sacrifice of certain effectiveness for a better patients’ satisfaction is worthwhile.

**Fig. 3:**
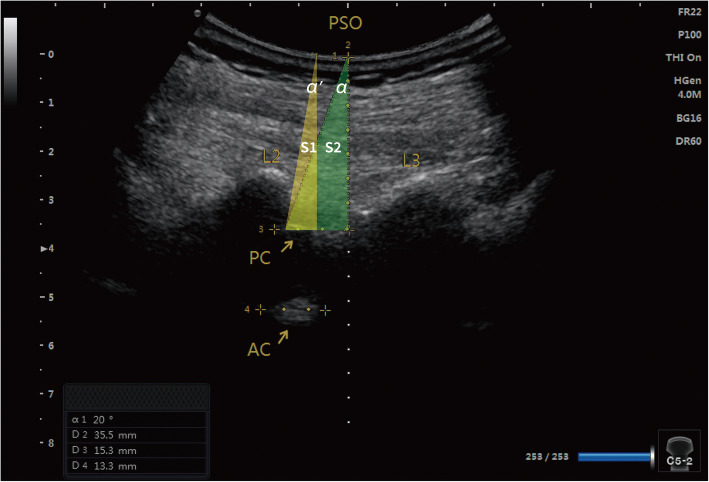
Comparison of operating spaces. The ultrasound image shows the L2-L3 interlaminar space in the parasagittal oblique (PSO) view. The posterior complex (PC) and anterior complex (AC) are shown at the same time. The modified preprocedural ultrasound-guided technique placed the upper edge of the inferior laminar at the center of the ultrasound screen and suggested a lower needle insertion point, which provided a larger cephalad angle (∠α), and a wider operation space (S2) than the previous technique which placed the PC and AC at the center of the screen (∠α’ and S1)

The higher first-pass success rate in the ultrasound group can be attributed to several modifications to previous ultrasound-assisted technique. First, accurately measured personalized insertion angles provided a better needle trajectory. Then the anesthesiologist used an aseptic protractor to guide the puncture. Previous studies have suggested 10–15° medial and cephalad angles during the puncture [28]. However, in practice, these angulations are estimated based on personal judgment. The results (Table 3) showed that for most cases (70%), the actual cephalad angle discrepancies were within 5°. Indeed, the first pass was also achieved in these cases. For the medial angle, 80% of the cases showed a discrepancy within 5°. These results demonstrated that the suggested angles could provide reasonable guidance. Second, for elderly patients with hip fractures, limitations associated with patient positioning may have led to a narrow interlaminar space. The current study placed the upper edge of the inferior lamina at the center of the screen to obtain a lower needle insertion point and a larger cephalad angle, resulting in a wider operating space for the puncture (Fig. 3). Previous studies have often placed the posterior and anterior complex at the center of the screen, and identified the needle insertion point by skin-marking the midpoint of the probe at that time [24, 29], thereby resulting in a relatively limited operating space (Fig. 3). Similar studies involving elderly patients also indicated that the first-pass success rate in the ultrasound group was higher than that reported in a study conducted by Park et al. [24], and a higher first-attempt rate was achieved compared with that reported in a study conducted by Geng et al. [25]. These favorable results could also be contributed by the modifications to the previous ultrasound-assisted technique.

In the current study, a successful first pass was not always accomplished. In most circumstances, this was because of bony contact, most frequently with the inferior laminar. Therefore, needle redirection and more needle passes were needed for a successful puncture. In five cases, the actual cephalad angle exceeded the maximum suggested angle (∠α) measured by the ultrasound image; this may have been explained by the deviation of the insertion point. If the marked needle insertion point was lower than the ideal point, the needle encountered the inferior laminar, and a larger cephalad angle was needed.

There is a difference in successful puncture interspace level between two groups. More patients achieved successful puncture at L2-L3 in landmark-guided group while L3-L4 in ultrasound-assisted group. The possible explanation is: in landmark-guided group, the anesthesiologist may not be absolutely sure about the identification of the intervertebral space. For safety concern, the anesthesiologist may intentionally choose a lower intervertebral space for needle insertion, resulted in a large proportion of L3-L4 level. In this case, the choice of the anesthesiologist is purely based on personal experiences and preferences. In the ultrasound-assisted group, the intervertebral space was identified by ultrasound scan, which is relatively more accurate. The ultrasound image indicated the L2-L3 level had better quality and were more suitable for needle insertion. In this case, this is not a choice but rather a more precise point derived from imaging results. When ultrasound scan showed the L3-L4 level had same image quality with L2-L3, the anesthesiologist would prefer L2-L3 for insertion according to surgery requirement.

Some procedural adverse reactions were observed in current study. Unintentional dural puncture occurred in three cases, possibly because the degenerative disc disease, ligament calcification, and stenosis of the spinal canal in elderly patients made it difficult to identify the tissue layer and control the force to perform the procedure [35]. Two cases in the landmark group required the use of alternative techniques, indicating that the variability of performance in the landmark group was relatively large compared with the ultrasound group. The differences in adverse reactions and postoperative complications were not statistically significant between the two groups. This might be attributed to the fact that the anesthesiologists in this study have ample experiences (> 15 years in average) in conducting CSE anesthesia.

Sub-group analysis for patients with scoliosis indicated that the modified ultrasound-assisted technique showed superiority in improving the first-pass success rate, reducing needle passes and attempts. The difference in total time was not statistically significant but longer time was consumed for puncture in the landmark-guided group which is invasive. In spite of patient satisfaction score was not statistically significant different in sub-group, which can be possibly explained by the limited number of cased for analysis, all patients in the ultrasound-assisted group scored 4–5.

However, we do acknowledge the limitations of this study. First, due to the nature of the study design, only the patients were blinded during the CSE anesthesia procedure. Second, measurement error was inevitable, even though the suggested cephalad and medial angles were measured by the same operator. Finally, in some cases, inaccuracy in the needle insertion point was unavoidable, as elderly patients often have loose and mobile skin.

## Conclusion

In conclusion, the modified preprocedural ultrasound-assisted CSE anesthesia technique with suggested needle insertion angulations and a more caudad needle insertion point can increase the first-pass and first-attempt success rate, reduce the puncture time and elevate patients’ satisfaction in elderly patients with hip fractures, especially in patients with scoliosis. We believe that it has clinical benefits for elderly patients with hip fractures.

## Supplementary information

**Additional file 1.**

## Data Availability

The datasets used and/or analyzed during the current study are available from the corresponding author on reasonable request.

## Abbreviations

AC: Anterior complex
ASA: American Society of Anesthesiologists
BMI: Body Mass Index
CI: Confidence Interval
CSE: Combined spinal-epidural
PC: Posterior complex
PSO: Paramedian sagittal oblique
SD: Standard deviation
TM: Transverse midline
