# Correction to: Landmark-guided versus modified ultrasound-assisted Paramedian techniques in combined spinal-epidural anesthesia for elderly patients with hip fractures: a randomized controlled trial

**DOI:** 10.1186/s12871-020-01183-8

**Published:** 2020-10-23

**Authors:** Bo Qu, Luying Chen, Yuling Zhang, Mengting Jiang, Caineng Wu, Wuhua Ma, Yuhui Li

**Affiliations:** 1 Guangzhou University of Chinese Medicine, Guangzhou, 510405 Guangdong China; 2 Department of Anesthesiology, The First Affiliated Hospital of Guangzhou University of Chinese Medicine, Guangzhou, 510405 Guangdong China

**Correction to: BMC Anesthesiol 20, 248 (2020)**

**https://doi.org/10.1186/s12871-020-01172-x**

Following publication of the original article [1], the authors reported two errors one in the Abstract section and one in the Result section.

In the Abstract section, the sentence “The median number of attempts was lower in ultrasound-assisted group (1 [1]) than landmark-guided group (2 [1, 2]), *P* < 0.001)” It should be corrected to “The median number of attempts was lower in ultrasound-assisted group (1 [1, 1]) than landmark-guided group (2 [1, 2]), P < 0.001).”

In the Result section, paragraph 7, the sentence “(Table 5) A subgroup analysis was conducted for 12 patients with scoliosis (Table 6).” should be corrected to “A subgroup analysis was conducted for 12 patients with scoliosis (Table 6).”

The original article has been updated.
