# Supplementary material for: Landmark-guided versus modified ultrasound-assisted Paramedian techniques in combined spinal-epidural anesthesia for elderly patients with hip fractures: a randomized controlled trial
Source: BMC Anesthesiol. 2020 Sep 28;20:248. doi: 10.1186/s12871-020-01172-x (PMC7523368; doi:10.1186/s12871-020-01172-x)
Supplement: Supplementary file 1 — Additional file 1. [file 12871_2020_1172_MOESM1_ESM.docx]

**Additional Table 1** Distribution of ultrasound image quality in both groups

|  |  | **Number of views**  **n (%)** |
| --- | --- | --- |
| Parasagittal oblique view | Good | 66 (82.5%) |
|  | Moderate | 14 (17.5%) |
|  | Poor | 0 (0%) |
| Transverse midline view | Good | 10 (12.5%) |
|  | Moderate | 35 (43.75%) |
|  | Poor | 35 (43.75%) |

Data are presented as n (%)
